# Older adults as Educators: Insights on Teaching, Learning and Well-Being Across Two Continents

**DOI:** 10.1093/geroni/igaf122.3253

**Published:** 2025-12-31

**Authors:** Giuliana Casanova, Margarida Cerqueira, Joyce Weil

**Affiliations:** University of Aveiro, Aveiro, Aveiro, Portugal; University of Aveiro, Aveiro, Aveiro, Portugal; Towson University, Towson, Maryland, United States

## Abstract

Older adults are often perceived as passive recipients of care, overlooking their capacity for meaningful contributions. Older adults also actively engage in lifelong learning, taking on the role of educators, sharing knowledge gained through their professional careers or hobbies with their peers at universities of the third age (U3As) in Portugal and in lifelong learning institutes (LLIs) in the USA. Still understudied in lifelong learning research, this study provides empirical insights into the experiences of older adults as educators. By analysing motivations, rewards, challenges, improvement opportunities and well-being outcomes, this research highlights the individual benefits and informs broader discussions on aging, education and best practices. This multiple case study includes a total of 35 retired older adults, aged 61 to 84. Semi-structured interviews and reflexive thematic analysis were used to explore key themes. Purpose driven engagement and passion for sharing knowledge emerged as primary motivations. Mutual learning, community and social connections as well as student development were the most valued rewards. Challenges related to teaching preparation, classroom dynamics and environmental constraints were identified. Marketing and outreach were noted as strategies for improvement. Emotional and cognitive health were identified as significant well-being outcomes. The Portuguese data reveal some similarities with the USA sample, particularly in relation to mutual learning, growth and classroom dynamics. This cross-cultural comparison provides a deeper understanding of the role of older adults as educators, highlighting the importance of their involvement in their lives and offering insights to enhance their engagement and contributions to education and their community.

